# Loss of disability-adjusted life years due to heat-related sleep disturbance in the Japanese

**DOI:** 10.1007/s41105-022-00419-z

**Published:** 2022-09-24

**Authors:** Tomohiko Ihara, Daisuke Narumi, Sanae Fukuda, Hiroaki Kondo, Yutaka Genchi

**Affiliations:** 1 Department of Environment Systems, Graduate School of Frontier Sciences, The University of Tokyo, 5-1-5 Kashiwanoha, Kashiwa, Chiba 277-8563 Japan; 2 Research Institute of Science for Safety and Sustainability, National Institute of Advanced Industrial Science and Technology (AIST), 16-1 Onogawa, Tsukuba, Ibaraki 305-8569 Japan; 3 Division of Social Engineering and Environmental Management, Graduate School of Environmental and Life Science, Okayama University, 3-1-1 Tsushimanaka, Kita-ku, Okayama, Okayama 700-8530 Japan; 4 Department of Health Science, Faculty of Health and Welfare, Kansai University of Welfare Sciences, 3-11-1 Asahigaoka, Kashiwara, Osaka 582-0026 Japan; 5 Japan Weather Association, 3-1-1, Higashi-ikebukuro, Toshima-ku, Tokyo, 177-6055 Japan; 6 Environmental Management Research Institute, National Institute of Advanced Industrial Science and Technology (AIST), Tsukuba, Japan, 16-1 Onogawa, Tsukuba, Ibaraki 305-8569 Japan

**Keywords:** Sleep disturbance, Climate change, Temperature, Health damage, DALY, PSQI

## Abstract

The purpose of this study was to quantify the sleep disturbances caused by climate change using disability-adjusted life years (DALY). The revised sleep quality index for daily sleep (SQIDS2), a self-administered questionnaire for daily sleep quality, was developed to assess daily sleep disturbances. This questionnaire referenced and simplified the Pittsburgh Sleep Quality Index (PSQI). This study was conducted in Nagoya City in August 2011 and 2012. Sleep quality was measured using SQIDS2 and PSQI. A total of 574 participants in 2011 and 710 in 2012 responded to the survey. The sleep disturbance prevalence calculated from the SQIDS2 score was correlated with the daily minimum temperature (*p* = 0.0067). This score increased when the daily minimum temperature was above 24.8 °C. When correcting for the PSQI score, DALY loss due to heat-related sleep disturbances in Nagoya City (population: 2,266,851) was estimated to be 81.8 years in 2012. This value was comparable to the DALY loss due to heatstroke. Sleep disturbance due to climate change was quantified using the DALY based on the PSQI. Legislators must recognize the critical impact of the damage caused by sleep disturbances due to high temperatures at night. Additionally, a daily minimum temperature of 25 °C should be the starting point when establishing a goal or guideline for nighttime temperature.

## Introduction

The temperature in urban areas has been rising persistently owing to both climate change and urban heat island phenomena. The recent report of the Intergovernmental Panel on Climate Change projects further temperature rises, increases in hot extremes, etc., under all future scenarios [1]. Such an increase in temperature will directly affect human health. When exposed to high temperatures, the human body increases the blood volume in the skin as well as the perspiration volume to dissipate heat. Insufficient heat dissipation leads to an increase in core body temperature, which may result in heatstroke [2]. Both sleep and body temperature are regulated by a circadian rhythm [3]. Sleep tends to occur when body temperature falls and ends when it rises. If the ambient temperature is not conducive to sleep, the sleep cycle is disturbed [4]. The World Health Organization (WHO) estimates that, because of climate change, the impact of direct heat exposure on health will become more significant than that of diseases such as dengue or malaria [5]. However, sleep disturbance is not included as one of the effects of direct heat exposure.

Several epidemiological studies have clarified the relationship between outdoor temperature and sleep disturbance [6]. Nastos and Matzarakis [7] described health emergency cases brought on by sleep disturbance, which were correlated with daily minimum temperatures. Obradovich et al. [8] quantified the relationship between insufficient sleep and minimum daily temperature. They estimated that there would be an increase in sleep disturbances related to future climate change. van Loenhout et al. [9] found that sleep disturbances increased by 11% for every 1 °C increase in outdoor temperature. Näyhä et al. [10] found that 32.4% of individuals experienced sleep disturbance during hot weather. Quante et al. [11] clarified the relationship between temperature and sleep duration using measurements recorded by accelerometers. Nonetheless, the numerical values shown in these studies did not compare deaths caused by direct heat exposure, as reported by the WHO. Therefore, the impact of sleep disturbances is poorly understood. Comparing sleep disturbances with other climate change-driven diseases requires quantifying the health damage brought on by sleep disturbances due to a rise in temperature. This damage can be quantified using a general health metric, such as the disability-adjusted life year (DALY).

Few studies have evaluated DALY for sleep disorder. The latest Global Burden of Disease (GBD) Study 2019 [12] only includes some diseases that include insomnia as their health status, such as infectious diseases, but excludes sleep disorders themselves. However, the 2004 GBD Study update [13] evaluated 3,623,000 years worldwide of DALY due to primary insomnia using its disability weight [14]. Deloitte Access Economics [15] evaluated DALY for obstructive sleep apnea, insomnia, and restless legs syndrome in Australia by estimating their disability weights based on the disability weight of insomnia from the GBD study. As a result, they estimated 190,000 years were lost due to sleep disorders in 2010. Hillman et al. [16] estimated the DALYs lost due to inadequate sleep in Australia, 2016–2017 by evaluating various conditions that occur because of inadequate sleep at 228,162 years. The assessment of DALYs due to sleep disturbances is limited to noise-induced sleep disturbances. WHO [17] estimated that the loss of DALY to environmental noise is 903,000 years for sleep disturbances in member states of the European Union and other Western European countries, based on a grade from 0 to 100 from epidemiological studies and the disability weight based on the GBD study and several previous studies. Similarly, Eriksson et al. [18] estimated that the DALYs due to sleep disturbances from road traffic and railroad noise were 22,218 years in Sweden. Begou and Kassomenos [19] estimated that the DALY due to sleep disturbances associated with exposure to road traffic noise in the urban complex in Thessaloniki-Neapoli in Greece ranged from 1247 to 3118 years, presuming a disability weight of 0.04 to 0.10. However, no study has assessed DALYs due to heat-related sleep disturbance, although Fukuda et al. [20] quantified its disability weight.

Based on studies that have quantified the relationship between sleep disturbance and outdoor temperature and studies that have been able to estimate DALYs due to sleep disturbance, we hypothesized that damage caused by heat-related sleep disturbance could be measured in DALYs. The aim of our study was to develop a method to measure DALY due to heat-related sleep disturbances and to measure DALY due to sleep disturbances caused by increases in outdoor temperature. It also compared the loss of life years due to sleep disturbance versus heatstroke.

## Methods

### Participants

**Fig. 1:**
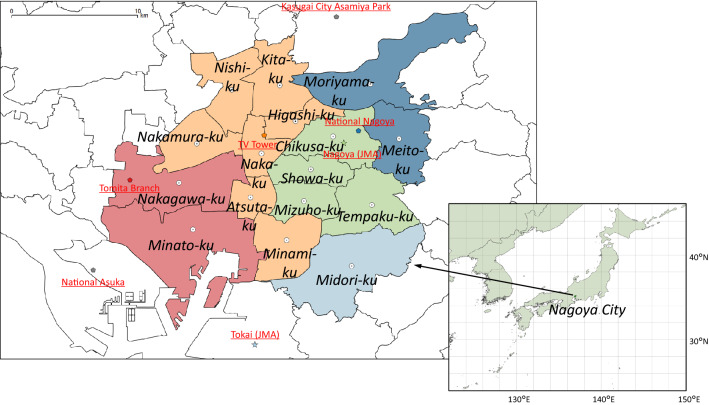
Survey target area (Nagoya City) and meteorological observation sites

We selected Nagoya City, the central city of the third metropolitan area in Japan, as our survey area. It is located at the center of Japan (Fig. 1). Nagoya City has been threatened by temperature rises due to climate change and its setting as a significant urban heat island. As a result, the annual average of outdoor temperature of Nagoya City has increased by 2.9 °C in the last 100 years [21]. Recently, the daily maximum temperature was higher than 35 °C for approximately 30 days in a year. This rise is the largest among Japan’s three major metropolitan areas (Tokyo, Osaka, and Nagoya).

The individuals who were registered as monitors for the survey company (INTAGE Research Inc., Tokyo, Japan) were included in our study. This ensured that various participants living in the city could continuously respond to our survey over an extended period. The online survey in Nagoya City was considered sufficiently representative, as the average personal internet usage rate in Japan was 79.1% in 2011. This rate was lower in rural areas than in urban areas, including Nagoya City [22]. Each adult participant was allowed to register by clicking on the company’s banner. The inclusion criteria were as follows: living in Nagoya City, not traveling during the survey period, and having access to a thermometer to measure room temperature. We recruited more than 550 participants in both years to ensure that the age (in increments of 10 years; from 20–29 years to 60–69 years and ≥ 70 years) and sex ratio of the respondents were equal to those of the population of Nagoya City.

The online survey was conducted in Nagoya City from August 2nd to the 11th (10 consecutive days) in 2011 and from July 31st to August 16th (9 days—Tuesdays, Wednesdays, and Thursdays—on 3 consecutive weeks) in 2012. We requested information regarding the respondents’ attributes (such as age and sex) and Pittsburgh Sleep Quality Index (PSQI) on the first survey day. Subsequently, we requested information using the revised sleep quality index for daily sleep (SQIDS2). We also requested information regarding the use of air conditioning on each survey day.

### Measures

One of the methods used to assess sleep disturbance to calculate DALY is using the PSQI.

DALY [23, 24] is a summary measure that combines time lost through premature death and time lived in states of less-than-optimal health. This is loosely referred to as “disability.” DALYs for a specific disease are calculated as the sum of the years of life lost (YLLs) from that cause and the years lived with a disability (YLDs) for people living in states of less-than-optimal health resulting from that specific cause (see formula (1)).

1 $$\mathrm{DALY}=\mathrm{YLL}+\mathrm{YLD}$$

2 $$\mathrm{YLL}=\mathrm{N}\times \mathrm{L}$$

3 $$\mathrm{YLD}=\mathrm{I}\times \mathrm{DW}\times \mathrm{L}.$$

In formula (2) for YLL, N is the number of deaths due to the cause, and L is the standard years of life lost at death. In formula (3) for YLD, I is the number of incident cases for the cause, DW is the disability weight for the cause (a weight factor that reflects the severity of the disease on a scale from 0, perfect health, to 1, death), and L is the average duration of the case until remission or death (years). DALY is widely used as a single health indicator that combines mortality and morbidity. The WHO quantifies health loss from hundreds of diseases, injuries, and risk factors in the Global Burden of Diseases (GBD) study. The WHO uses DALY to improve health systems. The GBD study 2019 highlighted that since 1990, there has been a marked shift toward a greater proportion of burden due to YLDs from non-communicable diseases and injuries [12].

PSQI [25, 26] is a widely used standardized measure to assess the subjective quality of sleep and generates a global score. Previous studies have shown that a global PSQI score of > 5 can distinguish between individuals with and without sleep disturbances [25, 27]. Since there is no direct mortality from heat-related sleep disturbances, we can set YLL to 0, similar to hearing loss [28] and low back pain [29]. The DALYs (YLDs) can be calculated for sleep disturbances by multiplying the prevalence of sleep disturbances obtained from the PSQI, the disability weight of sleep disturbances [20], and the duration of sleep disturbances.

The PSQI examines sleep quality for the past month; however, it cannot be correlated with daily temperature. Therefore, a self-administered questionnaire was developed to examine daily sleep quality by simplifying the PSQI—Sleep Quality Index for Daily Sleep (SQIDS) to correlate the daily temperature with sleep disturbances [30]. To reduce the number of questions, the survey questions of the original SQIDS did not coincide with the PSQI. In this study, the SQIDS was revised to be consistent with most of the survey questions in the PSQI (hereafter referred to as SQIDS2). Additionally, we used the PSQI on the first day of each survey to compare the PSQI and SQIDS2 scores. Using SQIDS2, we assessed the relationship between the daily prevalence of sleep disturbance and daily temperature. Furthermore, we were able to quantify the impact of temperature increases on sleep disturbance using DALY. We were then able to compare this impact with that of other illnesses.

We used outdoor temperature data from two observation sites of the Japan Meteorological Agency (JMA) (https://www.jma.go.jp/jma/menu/menureport.html). We also used three air pollution monitoring sites from the Ministry of the Environment of Japan (http://taiki-kankyo-aichi.jp/kanshi/realtime/). Nagoya City is divided into 16 wards (Fig. 1). The outdoor temperature of each respondent was defined as the temperature from the site closest to the population center of gravity of the ward where they lived.

### Data analysis

We calculated the PSQI global scores for the two first days and the SQIDS2 global scores for 19 days. The monthly and daily sleep disturbance prevalence was calculated by judging that the respondents who had a global score above 5.5 suffered from sleep disturbance, as per the cut-off point in the PSQI global score [25, 27].

We first examined the frequency distribution of age and sex. We used the Student’s *t* test and the Kruskal–Wallis one-way analysis to determine differences according to age and sex. The significance level was set at 5%.

We assessed the reliability of the PSQI and the SQIDS2 using Cronbach’s *α* [31] and McDonald’s *ω*_t_ [32]. The results of the PSQI survey and the first day of the SQIDS2 survey were used for our assessment. Cronbach’s *α* has been often used for assessing the reliability of the PSQI while McDonalds’s *ω*_t_ is shown as the best reliability indicator among 13 reliability indicators including Cronbach’s *α* [33] and is also used to assess PSQI [34]. Before calculating Cronbach’s *α* and McDonald’s *ω*_t_, we conducted exploratory factor analysis (EFA) and confirmatory factor analysis (CFA) to investigate the factor structures. First, we assessed the suitability for performing the factor analysis by calculating the Kaiser–Meyer–Olkin measure of sampling adequacy. It was assessed suitable that the Kaiser–Meyer–Olkin measure of sampling adequacy was greater than 0.6 [35]. The appropriate number of factors was then determined by parallel analysis, Velicer’s minimum average partial (MAP) test, and Bayesian information criterion (BIC). Using the appropriate numbers obtained, we completed the EFA using the minimum residual (minres) solution with the oblimin rotation to estimate the possible factor structures. To complement the EFA, we also performed the CFA using maximum likelihood estimation. We calculated the following parameters to evaluate model fit: the comparative fit index (CFI), the standardized root mean square residual (SRMR) and the root mean square error of the approximation (RMSEA). For the best fit models, we summarized the standardized regression weights for path (factor loadings on each factor) in figures. Among the possible factor structures, we decided that the structure that had the best parameters was the appropriate factor structure. For the above factor analysis, we used the packages psych version 2.2.5, GPArotation version 2022.4-1, lavaan version 0.6-11, and semPlot version 1.1.5 under R version 4.2.0.

We assessed the validity of SQIDS2 by assessing the correlation between the number of days of sleep disturbance determined by SQIDS2 and the determination of sleep disturbance by PSQI for the same respondent during the survey period. In our survey, PSQI assessed sleep during the month prior to the first day of the survey, while SQIDS2 assessed sleep on the day before the survey day. However, in previous Japanese PSQI survey conducted at the end of September, a climatically mild season of year, the prevalence of sleep disturbances ranged from 26.4 to 31.1% [36], suggesting that heat-related sleep disturbances were not a significant part of overall sleep disturbances. In addition, the sleeps assessed by PSQI and those by SQIDS2 existed in consecutive 1.5 months, and sleep disturbances were not considered to alter significantly. Therfore, if SQIDS2 determnations follow PSQI determinations, subjects who are determined to sleep disturbance many days by SQIDS2 must tend to be sleep distrubance determined by PSQI. The correlation was evaluated for each year.

Subsequently, a generalized linear model was used. This model illustrated the relationship between daily outdoor minimum temperature, *T*_min_, and sleep disturbance prevalence, sd. The sd was estimated by SQIDS2 using R version 3.5.2. A binomial distribution was assumed, and smoothed spline regression was applied.

4 $${\text{sd}}\sim s\left({T}_{\mathrm{min}}\right).$$

We used the daily minimum temperature as the prediction variable for the equation because the temperature of the bedroom at night that affects sleep quality is well correlated with the outdoor temperature at night [37] and two of the three previous studies [7–9] adopted the minimum outdoor temperature for their analysis.

We calculated the sleep disturbance prevalence based on the SQIDS2 for the previous month by applying regression (4) to the daily observed temperature data of the previous month. Regression (4) was adjusted by comparing the prevalence estimated by the PSQI and SQIDS2 for the same period (previous month).

Using the adjusted regression (4), the daily sleep disturbance prevalence can be calculated from the daily minimum outdoor temperature. The daily YLD loss was then calculated using the disability weight of sleep disturbance and assuming that the duration of sleep disturbance is one day. Furthermore, assuming YLL = 0, the DALY loss per day due to heat-related sleep disturbance was calculated. The DALY loss due to heat-related sleep disturbance for one year was calculated by performing the above operations for 365 days.

## Results

**Fig. 2:**
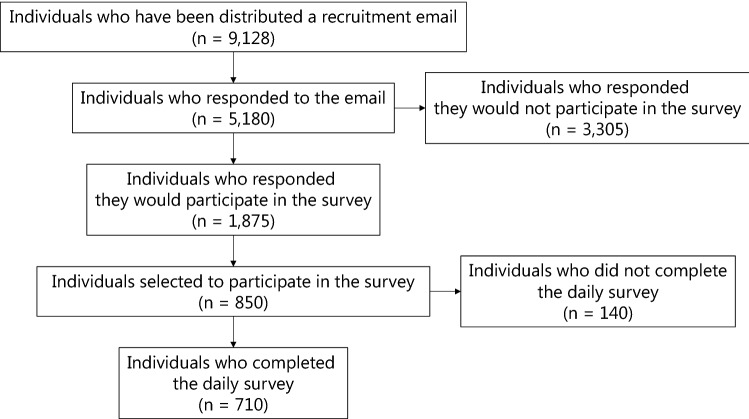
Flow diagram of the subjects

In the 2012 survey, the survey company distributed an email to recruit 9,128 adults from their online panel (Fig. 2). Of the 5,180 individuals who replied to the email, 1,875 participated in the survey. Eight hundred and fifty of these were finally selected to participate in the study. A total of 710 participants (83.5%) completed the daily survey. A total of 574 respondents participated in the 2011 survey. Although the 2011 survey was conducted using the same procedure as those in the 2012 survey, it did not provide detailed data. It should be noted that the 2011 survey and the 2012 survey were conducted independently. There are two independent cohort studies, not just one longitudinal study.

Age and sex ratios are presented in Table 1. The number of respondents aged ≥ 70 years was small—15 in 2011 and 26 in 2012. Except in the ≥ 70 years age group, the age and sex ratio of the respondents were representative of the population of Nagoya City. The valid response rate was the same as the response rate, since the online survey company requested that the respondents answer according to the prescribed format.

**Table 1:** Age and sex ratio of the respondents in the 2011 and 2012 surveys, including the 2010 population census

	20–29 years		30–39 years		40–49 years		50–59 years		60–69 years		≥ 70 years		Total	
2011 Survey														
Male	42	(7.3%)	52	(9.1%)	69	(12.0%)	61	(10.6%)	46	(8.0%)	12	(2.1%)	282	(49.1%)
Female	62	(10.8%)	63	(11.0%)	71	(12.4%)	56	(9.8%)	37	(6.4%)	3	(0.5%)	292	(50.9%)
2012 Survey														
Male	51	(7.2%)	86	(12.1%)	76	(10.7%)	72	(10.1%)	57	(8.0%)	15	(2.1%)	357	(50.3%)
Female	65	(9.2%)	72	(10.1%)	69	(9.7%)	99	(13.9%)	37	(5.2%)	11	(1.5%)	353	(49.7%)
2010 Population census														
Male	–	(7.6%)	–	(9.5%)	–	(8.8%)	–	(7.3%)	–	(8.1%)	–	(7.4%)	–	(48.9%)
Female	–	(7.3%)	–	(9.2%)	–	(8.4%)	–	(7.1%)	–	(8.4%)	–	(10.7%)	–	(51.1%)

The prevalence of sleep disturbance was estimated using the PSQI results (Table 2). The population-weighted average prevalence of sleep disturbance was 34.7% (95% confidence interval 32.1–37.3%). There were no significant differences between the survey years ($p=0.33$) or sexes ($p=0.17$), according to the *t* test. Nevertheless, there were significant differences between the age groups in the male population according to the Kruskal–Wallis one-way analysis of variance test ($p=0.0080$). The prevalence of sleep disturbance was higher in younger men than in older men. There were no significant differences between age groups in the female population ($p=0.79$).

**Table 2:** PSQI scores and prevalence of sleep disturbance among the respondents

	N	C1		C2		C3		C4		C5		C6		C7		Global		Sleep disturbance	
M																			
20–29	93	1.30 (1.17–1.43)		0.94 (0.77–1.10)		1.38 (1.19–1.56)		0.14 (0.04–0.24)		0.82 (0.72–0.91)		0.14 (0.02–0.26)		0.46 (0.32–0.60)		5.17 (4.72–5.63)		43.0% (32.9–53.1%)	
30–39	138	1.29 (1.18–1.40)		0.93 (0.77–1.09)		1.41 (1.27–1.54)		0.24 (0.14–0.34)		0.88 (0.78–0.97)		0.30 (0.15–0.46)		0.40 (0.28–0.52)		5.44 (4.95–5.93)		41.3% (33.1–49.5%)	
40–49	145	1.16 (1.07–1.25)		0.78 (0.64–0.91)		1.62 (1.49–1.75)		0.21 (0.12–0.31)		0.88 (0.80–0.96)		0.26 (0.13–0.39)		0.39 (0.27–0.51)		5.31 (4.85–5.77)		36.6% (28.7–44.4%)	
50–59	133	1.20 (1.09–1.30)		0.71 (0.56–0.86)		1.41 (1.27–1.56)		0.20 (0.10–0.29)		0.85 (0.76–0.94)		0.20 (0.09–0.32)		0.32 (0.21–0.43)		4.89 (4.46–5.31)		30.8% (22.9–38.7%)	
60–69	103	1.02 (0.93–1.11)		0.68 (0.52–0.84)		0.96 (0.81–1.11)		0.17 (0.08–0.27)		0.93 (0.85–1.01)		0.20 (0.07–0.34)		0.15 (0.07–0.22)		4.12 (3.71–4.53)		21.4% (13.4–29.3%)	
≥ 70	27	0.89 (0.65–1.13)		0.78 (0.44–1.11)		0.96 (0.64–1.29)		0.19 (− 0.05–0.42)		0.96 (0.80–1.13)		0.67 (0.19–1.15)		0.22 (0.03–0.41)		4.67 (3.74–5.59)		25.9% (9.1–42.8%)	
F																			
20–29	127	1.41 (1.29–1.53)		1.14 (0.96–1.32)		1.20 (1.04–1.37)		0.22 (0.12–0.32)		0.82 (0.72–0.91)		0.21 (0.09–0.34)		0.49 (0.34–0.63)		5.50 (4.99–6.01)		41.7% (33.1–50.3%)	
30–39	135	1.36 (1.23–1.48)		1.12 (0.95–1.28)		1.21 (1.06–1.36)		0.16 (0.09–0.24)		0.97 (0.88–1.06)		0.22 (0.09–0.35)		0.41 (0.29–0.54)		5.45 (4.99–5.92)		40.0% (31.7–48.3%)	
40–49	140	1.22 (1.13–1.32)		0.84 (0.69–0.99)		1.51 (1.38–1.64)		0.14 (0.06–0.22)		0.93 (0.85–1.01)		0.19 (0.08–0.31)		0.41 (0.29–0.54)		5.25 (4.83–5.67)		35.7% (27.7–43.7%)	
50–59	155	1.29 (1.19–1.39)		0.97 (0.82–1.12)		1.50 (1.38–1.63)		0.16 (0.08–0.24)		0.93 (0.85–1.01)		0.17 (0.07–0.27)		0.35 (0.26–0.45)		5.37 (4.98–5.77)		38.7% (31.0–46.4%)	
60–69	74	1.18 (1.04–1.31)		0.88 (0.67–1.08)		1.23 (1.05–1.40)		0.18 (0.08–0.27)		0.91 (0.80–1.01)		0.42 (0.19–0.65)		0.27 (0.16–0.39)		5.05 (4.41–5.70)		33.8% (22.9–44.6%)	
≥ 70	14	0.93 (0.68–1.18)		1.00 (0.64–1.36)		1.00 (0.59–1.41)		0.29 (− 0.15–0.72)		1.00 (0.79–1.21)		0.29 (− 0.15–0.72)		0.50 (0.10–0.90)		5.00 (3.91–6.09)		28.6% (4.0–53.1%)	
Unweighted	1284	1.24 (1.20–1.27)		0.90 (0.85–0.95)		1.35 (1.30–1.40)		0.19 (0.16–0.21)		0.90 (0.87–0.92)		0.24 (0.19–0.28)		0.37 (0.33–0.41)		5.18 (5.03–5.32)		36.3% (33.7–38.9%)	
Weighted	–	1.18 (1.15–1.22)		0.90 (0.85–0.95)		1.28 (1.23–1.32)		0.19 (0.16–0.23)		0.91 (0.88–0.94)		0.27 (0.23–0.32)		0.37 (0.33–0.41)		5.11 (4.97–5.25)		34.7% (32.1–37.3%)	

C1–C7 and Global are the scores for each component of the PSQI and the PSQI global score, respectively. Values in parentheses indicate a 95% confidence interval. Unweighted value means simple summary; weighted value is the result considering the age and sex structure of the population

**Fig. 3:**
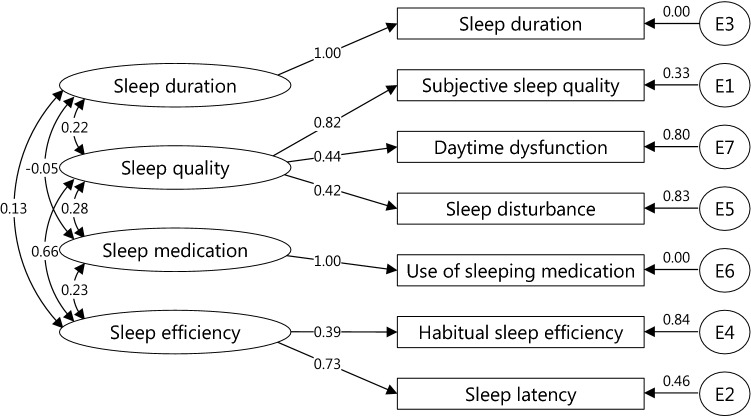
Standardized regression weights for paths associated with the best fit model for PSQI

**Fig. 4:**
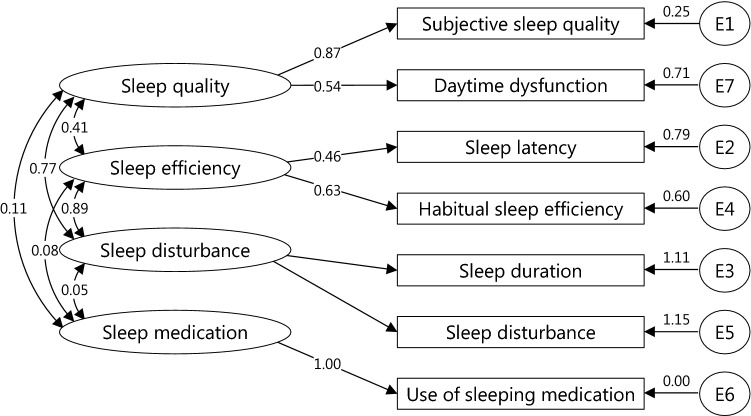
Standardized regression weights for paths associated with the best fit model for SQIDS2

Previous studies have suggested that the removal of the C6 (use of sleeping medication) improved the Cronbach’s *α* when targeting healthy subjects [38]. Therefore, cases in which the C6 was removed were assessed, which are indicated by the numbers in parentheses in the following description. The Kaiser–Meyer–Olkin measure of PSQI was 0.68 (0.66). The parallel analysis, MAP, and BIC revealed that the number of factors of PSQI was 4, 1, and 3 (3, 1, and 2), respectively. Based on the factor structures estimated by the exploratory factor analysis, a series of confirmatory factor analyses were performed (Table 3). Among the models, the P4 model showed reasonable fit: CFI = 0.893, SRMR = 0.047, and RMSEA = 0.089. The standardized regression weights for the paths associated with the model P4 are shown in Fig. 3. The Kaiser–Meier–Olkin measure for SQIDS2 was 0.59 (0.59). Parallel analysis, MAP, and BIC revealed that the number of factors of PSQI was 4, 1, and 3 (3, 1, and 2), respectively. A series of confirmatory factor analysis was conducted (Table 4). Among the models, model S4 indicated an adequate fit: CFI = 0.909, SRMR = 0.042, and RMSEA = 0.084. The standardized regression weights for the paths associated with model S4 are shown in Fig. 4. The Cronbach’s *α* was 0.57 (0.57) for PSQI and 0.52 (0.54) for SQIDS2 whereas the McDonald’s *ω*_t_ was 0.78 if the number of factors = 4 (0.73 if the number of factors = 3) for PSQI and 0.51 if the number of factors = 3 (0.72 if the number of factors = 3) for SQIDS2.

**Table 3:** Models for PSQI and using confirmatory factor analysis (*n *= 1284)

Models	*χ*^2^	df	*p* value	CFI	SRMR	RMSEA
Model P1: 1 factor, 7 components	167.440	14	0.000	0.836	0.057	0.092
Model P2: 2 factors, 7 components (F1: 1, 2, 4, 5, 6, and 7; F2: 3)	167.440	14	0.000	0.836	0.057	0.092
Model P3: 3 factors, 7 components (F1: 1, 6, and 7; F2: 2, 4, and 5; F3: 3)	122.347	12	0.000	0.882	0.049	0.085
Model P4: 4 factors, 7 components (F1: 1, 5, and 7; F2: 2 and 4; F3: 6; F4: 3)	110.749	10	0.000	0.893	0.047	0.089
Model P(-6)1: 1 factor, 6 components	134.226	9	0.000	0.850	0.057	0.104
Model P(-6)2: 2 factors, 6 components (F1: 1, 2, 4, 5, and 7; F2: 3)	134.226	9	0.000	0.850	0.057	0.104
Model P(-6)3: 3 factors, 6 components (F1: 1, 5, and 7; F2: 2 and 4; F3: 3)	95.447	7	0.000	0.894	0.051	0.099

*df* degree of freedom, *CFI* comparative fit index, *SRMR* standardized root mean square residual, *RMSEA* root mean square error of approximation

**Table 4:** Models for SQIDS2 and using confirmatory factor analysis (*n *= 1284)

Models	*χ*^2^	df	*p* value	CFI	SRMR	RMSEA
Model S1: 1 factor, 7 components	301.243	14	0.000	0.682	0.079	0.126
Model S2: 2 factors, 7 components (F1: 1, 3, and 7; F2: 2, 4, 5, and 6)	201.505	13	0.000	0.791	0.059	0.106
Model S3: 3 factors, 7 components (F1: 1, 6, and 7; F2: 2, 4, and 5; F3: 3)	200.747	12	0.000	0.791	0.059	0.111
Model S4: 4 factors, 7 components (F1: 1 and 7; F2: 2, 4 and 6; F3: 5; F4: 3)	90.912	9	0.000	0.909	0.042	0.084
Model S(-6)1: 1 factor, 6 components	290.595	9	0.000	0.683	0.089	0.156
Model S(-6)2: 2 factors, 6 components (F1: 1, 3, 4, 5, and 7; F2: 2)	290.595	9	0.000	0.683	0.089	0.156
Model S(-6)3: 3 factors, 6 components (F1: 1 and 7; F2: 2 and 4; F3: 3 and 5)	219.558	8	0.000	0.762	0.078	0.144

*df* degree of freedom, *CFI* comparative fit index, *SRMR* standardized root mean square residual, *RMSEA* root mean square error of approximation

**Fig. 5:**
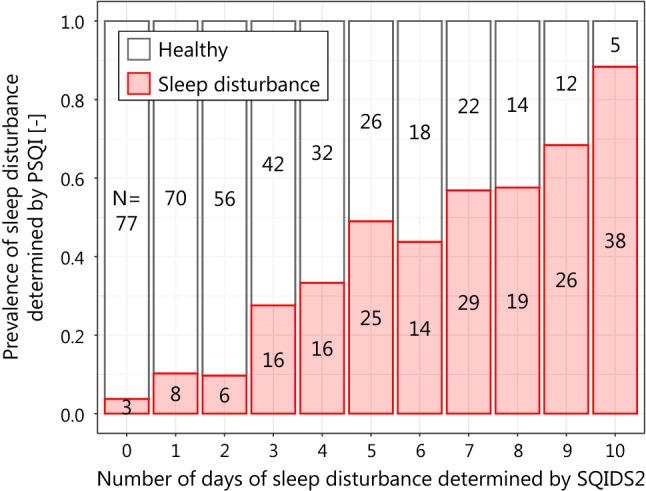
Relationship of sleep disturbance determinations between SQIDS2 and PSQI in 2011

**Fig. 6:**
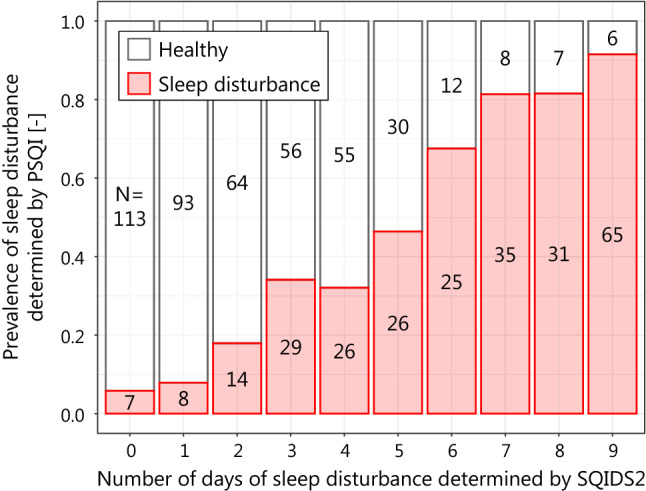
Relationship of sleep disturbance determinations between SQIDS2 and PSQI in 2012

The relationship of sleep disturbance determination between SQIDS2 and PSQI for 2011 and 2012 is shown in Figs. 5 and 6. The horizontal axis is the number of days of sleep disturbance determined by SQIDS2, and the vertical axis is the prevalence of sleep disturbance determined by PSQI among these people. The coefficient of determination was 0.988 for 2011 and 0.991 for 2012.

**Fig. 7:**
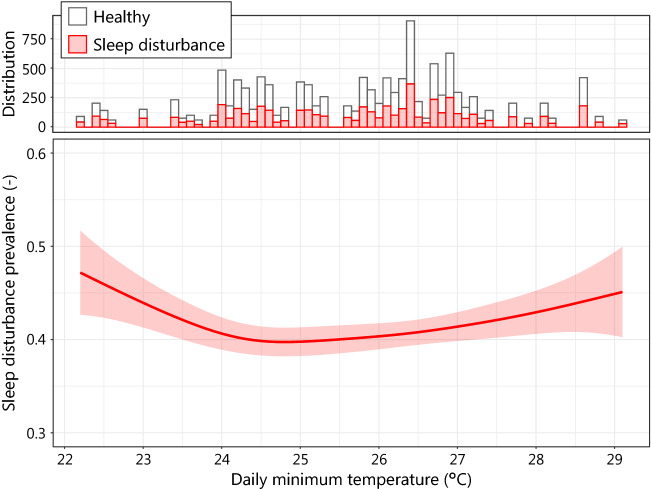
Relationship between daily minimum temperature and sleep disturbance prevalence calculated from SQIDS2 scores

We analyzed the relationship between the daily minimum temperature and prevalence of sleep disturbance calculated from the SQIDS2 scores (Fig. 7). The sleep disturbance prevalence calculated using SQIDS2 correlated with the daily minimum temperature ($p=0.0067$). The prevalence increased when the daily minimum temperature was above 24.8 °C. Hereafter, the prevalence of excess sleep disturbance on a day with a minimum temperature above 24.8 °C is defined as heat-related sleep disturbance prevalence.

The sleep disturbance prevalence ${\text{s}}{\text{d}}_{\text{SQIDS2}}$ calculated from regression (4) for the PSQI target periods (past month) in 2011 and 2012 correlated with that estimated ${\text{s}}{\text{d}}_{\text{PSQI}}$ from the PSQI scores in the regression curve through the origin. The correlation was calculated as follows,

$${\text{s}}{\text{d}}_{\text{PSQI}}=0.839{\text{s}}{\text{d}}_{\text{SQIDS2}}.$$

**Fig. 8:**
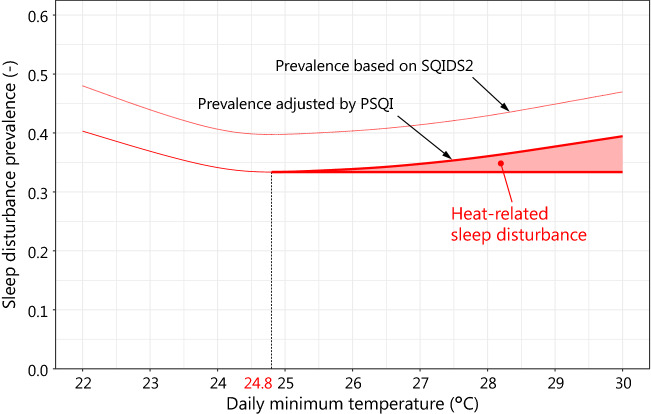
Relationship between sleep disturbance prevalence calculated from the regression model based on SQIDS2 scores and those estimated from PSQI scores in 2011 and 2012

However, regression (4) overestimated the prevalence of sleep disturbances by 19% compared to PSQI. Therefore, the prevalence of sleep disturbance based on SQIDS2 must be corrected. This is necessary when evaluating actual sleep disturbances (Fig. 8), which can be assessed using DALY.

The population of Nagoya City was 2,266,851 in 2012 [39]. The annual total number of individuals who experienced heat-related sleep disturbance based on SQIDS2 was estimated at 355,000. This was calculated using the daily minimum temperature for each day in 2012 and the regression model. The disability weight for sleep disturbance was 0.1 [20]. The duration of sleep disturbance was set to 1 day (as prevalence was counted every day), and the conversion factor from SQIDS2 to PSQI was 0.839 (as SQIDS2 overestimated sleep disturbance prevalence by 19%). Therefore, the DALY loss due to heat-related sleep disturbance in Nagoya City was estimated to be 81.8 years in 2012.

## Discussion

In this study, we developed SQIDS2 and surveyed the quality of sleep of residents using PSQI and SQIDS2 to quantify the relationship between daily minimum temperature and sleep disturbance.

The number of respondents aged ≥ 70 years was small in 2011 and 2012. This was due to the low use of the Internet among the elderly population [22]. Doi et al. [36] conducted a postal survey of the PSQI throughout Japan in September 1997 and found that the prevalence of sleep difficulty ranged from 20.9 to 44.3% (30.0% when simply averaged by age and sex). Compared with the large sample size of patients in their 60 s or younger, the frequency of sleep difficulties in this study was 9.4% higher (the study by Doi et al. was 27.4%, and our study was 36.3%). Differences in C1–C7 scores (this study—Doi et al.) were 0.30, 0.10, 0.39, 0.05, 0.19, 0.12, and − 0.28, respectively, with C3 (sleep duration) being the largest. A Japanese time use survey [40] showed that sleep decreased by 0.13 h in people aged 20–60 years, from 7.59 h in 1996 to 7.46 h in 2011. It is possible that shorter sleep duration has increased the prevalence of sleep disturbance estimated from a PSQI survey.

Exploratory and confirmatory factor analyses indicated that PSQI in this study has a four-factor structure: sleep duration, sleep quality, sleep medication, and sleep efficiency. Buysse et al. [25] assumed that the PSQI had a one-factor structure. However, in previous studies on healthy subjects, the PSQI showed a multifactorial structure [34, 38, 41–44]. Since calculating Cronbach’s *α* premises a one-factor structure [45], it is considered that low Cronbach’s *α* does not immediately imply a lack of internal consistency [46]. Previous PSQI surveys on healthy subjects showed low Cronbach’s *α* [38, 47–50], including this study. However, PSQI in this study showed that McDonald’s *ω*_t_ was greater than 0.7, so it can be considered internally consistent. Exploratory and confirmatory factor analyses showed that SQIDS2 has a four-factor structure: sleep quality, sleep efficiency, sleep disturbance, and sleep medication. Because it was not a one-factor structure, the Cronbachs *α* value was low. The McDonalds *ω*_t_ value was also low. This is likely due to the fact that the use of sleeping medication was asked of healthy subjects on a daily basis. In fact, only 7.4% of respondents did not have a C6 of 0. When removing the C6, the McDonalds *ω*_t_ value was greater than 0.7. This result corresponds to previous PSQI studies on healthy subjects [38].

SQIDS2 cannot be validated by the distinction between clinical and non-clinical subjects because daily sleep disturbance is not diagnosed. However, the accumulation of daily sleep disturbance must be associated with to sleep disturbance. Our analysis showed a high coefficient of determination between the number of days of sleep disturbance determined by SQIDS2 and the prevalence of sleep disturbance determined by PSQI. Therefore, SQIDS2 is considered to follow PSQI well.

The regression of SQIDS2 sleep disturbance to daily temperature [regression (4)] overestimated sleep disturbance by 19% compared with the PSQI. This is due to the difference in targeted sleep (SQIDS2 for the previous day or PSQI for the previous month).

There are three similar studies [7–9]. Here, the incremental number of sleep disturbances for a unit temperature increase is referred to as the beta value. In a year-round survey of 766,761 adults in the United States, Obradovich et al. [8] used the questions 'During the past 30 days, for about how many days have you felt you did not get enough rest or sleep?' as an indicator of sleep disturbance and monthly mean daily minimum temperature anomaly as an indicator of temperature and found a beta of 0.41%. Similarly, in a survey of 826 and 1400 admissions to sleep disturbance throughout the year in Athens, Greece, Nastos and Matzarakis [7] used daily hospital psychiatric emergency unit admissions and 10-day mean daily minimum temperature and found a beta of 2.1% in 1989 and 1.5% in 1994. In a survey of 113 older adults from May to August in Arnhem and Groningen, the Netherlands, van Loenhout [9] used the question “Did you have the symptom ‘Not sleeping well’ due to heat during the past week?” and the weekly mean daily mean temperature and found a beta of 11%. This study found a beta of 0.58%. Three of these studies, except for the study by van Loenhout et al. [9] showed that the beta value was approximately 0.5–2%, although each of these studies used different indicators and meteorological data to calculate sleep disturbance. Although van Loenhout et al. reported a beta of 11%, their results are reasonable, because they limited their analysis to “heat-related” sleep disturbances. Similar to the data by Obradovich et al., our results showed the damaging effects of sleep disturbance. Obradovich et al. did not consider the existence of an optimum temperature in their study. This difference can be explained by two reasons. First, Obradovich et al. conducted their survey throughout the year, whereas this study was conducted only in the summer. It is well known that sleep disturbance changes are seasonal [51]. This seasonal effect partially includes temperature fluctuations; however, further analysis is required. The second reason is the differences in the indicators examined. Variations in sleep disturbance caused by temperature changes were measured using different methods. Therefore, a single unified method is required for future studies.

Cool outdoor air cools the indoor air of residential houses heated during the day by ventilation through windows and heat transfer through sidewalls and roofs during summer nights in Japan. In a previous survey, a strong correlation was observed between outdoor temperature and bedroom temperature, with the minimum bedroom temperature being 3.4 °C higher than the minimum outdoor temperature in summer in Nagoya [37]. Since the mean comfort temperature in the bedroom in summer in Japan is 26.4 to 27.1 °C [52], the bedroom temperature is considered to have exceeded the comfort temperature when the outdoor temperature exceeds 24.8 °C.

The use of air conditioning in residential houses in Nagoya City was as high as 96.6% in 2014 [53]. However, a previous survey on the sleeping environment of elderly people in Tokyo, Japan, found that many people install air conditioners in their bedrooms, but they sleep with little or no air conditioning in summer [54]. Installing and appropriately using air conditioning is one solution to reduce heat-related sleep disturbances. However, it is also important to reduce outdoor temperatures, as some residents are unable to install or use air conditioning for various reasons [54]. Promising measures to reduce urban heat islands, such as increasing the albedo of urban surfaces and installing vegetated green spaces, have limited effect on reducing nighttime temperature [55], because there is no solar radiation at night. Since the atmosphere is relatively stable at night, anthropogenic heat reduction measures, such as the installation of air-source heat pump water heaters [56], will play an important role in reducing the outdoor temperature to 24.8 °C.

**Fig. 9:**
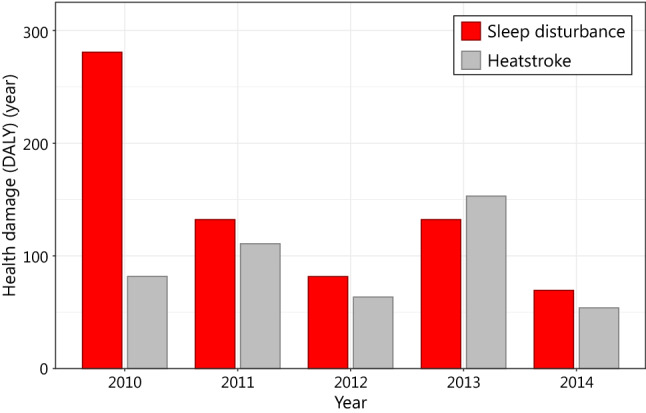
Comparison of health damage due to heat-related sleep disturbance and heatstroke in Nagoya City from 2010 to 2014

Death due to heatstroke is an unusual heat-related event. In Nagoya, five people died from heatstroke in 2012. Their DALY was estimated to be 63.54 years [57] when using the vital statistics table for Nagoya City [58] (their DALY was estimated to be 32 years for a lifespan of 85 years). From 2010 to 2014, an average of seven deaths due to heatstroke were reported each year. The annual DALY loss for these individuals was 92.66 years (59.8 years for a lifespan of 85 years). In Nagoya, in 2012, the loss of DALY due to heat-related sleep disturbance was estimated at 81.8 years. From 2010 to 2014, the annual estimate ranged from 69.4 to 280.6 years (Fig. 9). Sleep disturbance is currently not considered relevant, although the problems it causes are comparable to those caused by heatstroke.

Limitations of this study include the damage function, which was developed only for summer. This study did not analyze the use of air conditioners, and it is unclear how exactly the use of air conditioners may have reduced heat-related disturbance. The mental health of the participants was not investigated. The sleep disturbance target was limited to adults registered on a web-based platform. Also, the target population may be too small to be evaluated using DALYs for heat-related sleep disturbances and heatstroke. Furthermore, although this study simply analyzed daily sleep, in reality, the 10 or 9 days of sleep of the same subject are not independent but correlate with each other. Multilevel analysis [59] will analyze sleep data nested within subjects more precisely.

Nonetheless, a health metric for non-fatal diseases is critical. This study developed SQIDS2 by referring to PSQI, which distinguishes sleep disturbances, and quantified for the first time the damage caused by heat-related sleep disturbances using DALY, a health metric comparable to those of other diseases. By quantifying the damage caused by sleep disturbance using the same metrics as that caused by heatstroke, legislators can recognize the damage caused by sleep disturbance due to high nocturnal temperatures. A daily minimum temperature of 25 °C should be the starting point when establishing regulations for a nighttime temperature. Large population surveys, including children and older adults, are necessary to adequately contribute toward more appropriate policies. In the future, it will also be essential to study sleep in other seasons and analyze the effects of countermeasures, such as air conditioning, using multilevel analysis.
